# Anti-endotoxin immunity in abdominal sepsis patients

**DOI:** 10.1186/cc10371

**Published:** 2011-10-27

**Authors:** O Butyrsky, V Starosek

**Affiliations:** 1 Department of Surgery, Crimean Medical University, Simferopol, Ukraine

## Introduction

Anti-endotoxin immunity (AEI) has many biological effects but the problem of conjugation and elimination of lipopolysaccharides (LPS) in peritonitis patients is not discussed. We investigated the role of IgA, IgM and IgG in peritonitis and their association with humoral immunity (HI).

## Methods

We investigated 33 patients (male:female = 25:8) with abdominal sepsis (total peritonitis in appendicitis, perforated duodenal ulcer, pancreonecrosis). Anti-endotoxin (AE) antibodies (anti-LPS-IgA, anti-LPS-IgM, anti-LPS-IgG) were determined by original modification of hard-phase immunoenzyme analysis. *Escherichia coli K30 *LPS was used as antigen for AE antibody detection. The level of general immunoglobulin was determined by the microturbidimetric method with human monospecific sera to IgG, IgA and IgM. All data were compared with healthy donors (99 patients).

## Results

A high level of AEI and HI was determined in 24% of patients who recovered rapidly without complication after surgery, discharged in 9 to 10 days. This was confirmed by clinical data (normalization of body temperature, peristalsis, spontaneous stool) by 4 to 5 days. A low level of AEI and HI was found in 42% of patients who recovered slowly; in a favorable course of peritonitis, the increase of parameters was marked by 8 to 10 days; in several with suppuration of wounds, discharge was in 14 to 16 days. A few patients with a low level of immunity against the background of abdominal sepsis required therapy with sandoglobulin H that was accompanied with a sharp positive change of a postoperative course of peritonitis and an increase of immunity indices. See Table 1. An evident decrease of AE antibodies may be a background for translocation of endotoxin from the intestine to the portal and systemic circulation. Disorder of AE mechanisms of endotoxin conjugation may activate other mechanisms of neutralization (endotoxin-conjugating protein) that stimulate CD14-receptor structures and mechanisms of active production of proinflammatory cytokines and starting systemic inflammatory response syndrome.

**Table 1:** 

			AEI						HI			
		
	IgA		IgG		IgM		IgA		IgG		IgM	
						
	Before surgery	After surgery	Before surgery	After surgery	Before surgery	After surgery	Before surgery	After surgery	Before surgery	After surgery	Before surgery	After surgery
Peritonitis patients	0.28 ± 0.01, *P *< 0.05	0.45 ± 0.02	0.12 ± 0.01, *P *< 0/01	0.13 ± 0.02	0.21 ± 0.03, *P *< 0/05	0.29 ± 0.04	2.26 ± 0.16, *P *> 0.5	2.77 ± 0.18	10.1 ± 0.47, *P *> 0.5	10.98 ± 0.5	1.39 ± 0.11, *P *< 0.05	1.56 ± 0.12
Donors	0.35 ± 0.05		0.16 ± 0.01		0.33 ± 0.05		2.21 ± 0.08		10.54 ± 0.242		1.66 ± 0.06	

## Conclusion

Abdominal sepsis patients are determined dysfunction of AEI (decrease of AE IgM and IgG). Successful treatment of peritonitis is accompanied with normalization of the IgM and IgG concentration and an increase of IgA above standard. Dynamics of AE antibodies may be a marker of the clinical course and forecast of abdominal sepsis. Comparative analysis of HI and AEI demonstrates parallelism of the dynamic concentration of immunoglobulins during treatment.

